# Misfortunes never come alone: melanoma and ulcerative colitis after biologic therapy in a psoriatic patient – a case report and literature review

**DOI:** 10.3389/fimmu.2025.1651517

**Published:** 2025-08-26

**Authors:** Aleksandra Wiktoria Bratborska, Magdalena Jałowska, Monika Bowszyc-Dmochowska, Michał J. Kowalczyk, Kinga Adamska

**Affiliations:** ^1^ Department of Dermatology and Venereology, Poznan University of Medical Sciences, Poznan, Poland; ^2^ Doctoral School, Poznan University of Medical Sciences, Poznan, Poland; ^3^ Department of Dermatology, Poznan University of Medical Sciences, Poznan, Poland; ^4^ Cutaneous Histopathology and Immunopathology Section, Department of Dermatology, Poznan University of Medical Sciences, Poznan, Poland

**Keywords:** psoriasis, biologics, biologic therapy, targeted therapy, skin inflammation, autoinflammatory skin diseases, autoinflammatory dermatoses

## Abstract

Psoriasis affects approximately 2% of the global population, with plaque psoriasis being its most prevalent manifestation. Management strategies range from topical therapies for mild cases to systemic interventions for moderate to severe cases. Those include immunosuppressive drugs and biological agents, which are novel therapeutic options that have revolutionized psoriasis management. Patients with psoriasis have a higher risk of developing other autoinflammatory diseases, as well as cancer. In addition, both classic immunosuppressive agents and biologics carry a risk of malignancies. We present a case of a 36-year-old male with a long history of plaque psoriasis and psoriatic arthritis. He was undergoing treatment with secukinumab and also had a history of therapy with infliximab. During the treatment course, the patient noted a progressively enlarging pigmented lesion on his left calf, which was subsequently diagnosed as superficial spreading melanoma. Following this diagnosis, he was informed about the potential oncological risks associated with biological therapy and switched to acitretin and methotrexate. Over two years later, he was diagnosed with ulcerative colitis (UC) after endoscopic evaluation. No melanoma recurrence has been observed to date. Our case report demonstrates the difficulties in managing malignancies in patients receiving biologic therapy, highlighting the necessity for careful medical assessment and examination of patients receiving those types of drugs. In cases of cancer coexistence, a multidisciplinary approach is crucial for the sufficient treatment of both oncological and autoinflammatory diseases. Additionally, medical professionals should be aware of the increased risk of developing other autoinflammatory conditions in individuals with psoriasis.

## 1 Introduction

Psoriasis represents a significant global health issue, affecting approximately 2% of the global population. The condition is prevalent among both men and women across all age groups. Nonetheless, analyses of its geographical distribution indicate a notably higher incidence in Northern and Western Europe (1). Psoriasis presents in various forms, with plaque psoriasis representing over 80% of cases and therefore, the most prevalent subtype (2). Over 30% of patients develop chronic inflammatory arthritis, specifically psoriatic arthritis (PsA), which, unless treated appropriately, ultimately results in joint destruction (3).

Topical therapy is typically reserved for patients who experience only mild psoriasis. A wide range of topical agents may be employed, including corticosteroids, calcineurin inhibitors, keratolytic creams, or vitamin D analogs (4). In addition, patients with mild manifestations may also benefit from targeted phototherapy. For those with moderate to severe psoriasis, systemic interventions are essential to manage the condition. These include phototherapy, oral medications, and biologic drugs. Traditional systemic agents comprise acitretin, ciclosporin, methotrexate, and apremilast (5). Biologic therapy represents an innovative and promising approach, employing mechanisms such as tumor necrosis factor (TNF)-α inhibition and targeting specific interleukins, including interleukin (IL)-17 and IL-23 inhibitors, among others (2, 6).

Immunosuppressive agents are an indispensable treatment option for patients with autoinflammatory diseases, including psoriasis. Nevertheless, they have been associated with an increased risk of certain neoplasms (7). Melanoma, being significantly influenced by immune cells, is classified as an immunogenic tumor (8). As a result, immunosuppressive and immunomodulating agents are said to affect melanoma development, prognosis, and recurrence (9).

Interestingly, evidence suggests a higher risk of malignancies in patients with psoriasis. These include squamous cell carcinoma (SCC), basal cell carcinoma (BCC), carcinomas of the digestive system, lymphomas, as well as lung or bladder cancer (10, 11). The increased malignancy risk is suggested to be a result of the combination of chronic low-grade inflammation associated with the disease, immunological modulation by antipsoriatic drugs, as well as certain harmful habits of patients suffering from the burden of the disease (12, 13). These include alcohol abuse, smoking, unhealthy diet, and minimal or even complete lack of physical activity (14–16).

## 2 Case presentation

**Figure 1:**
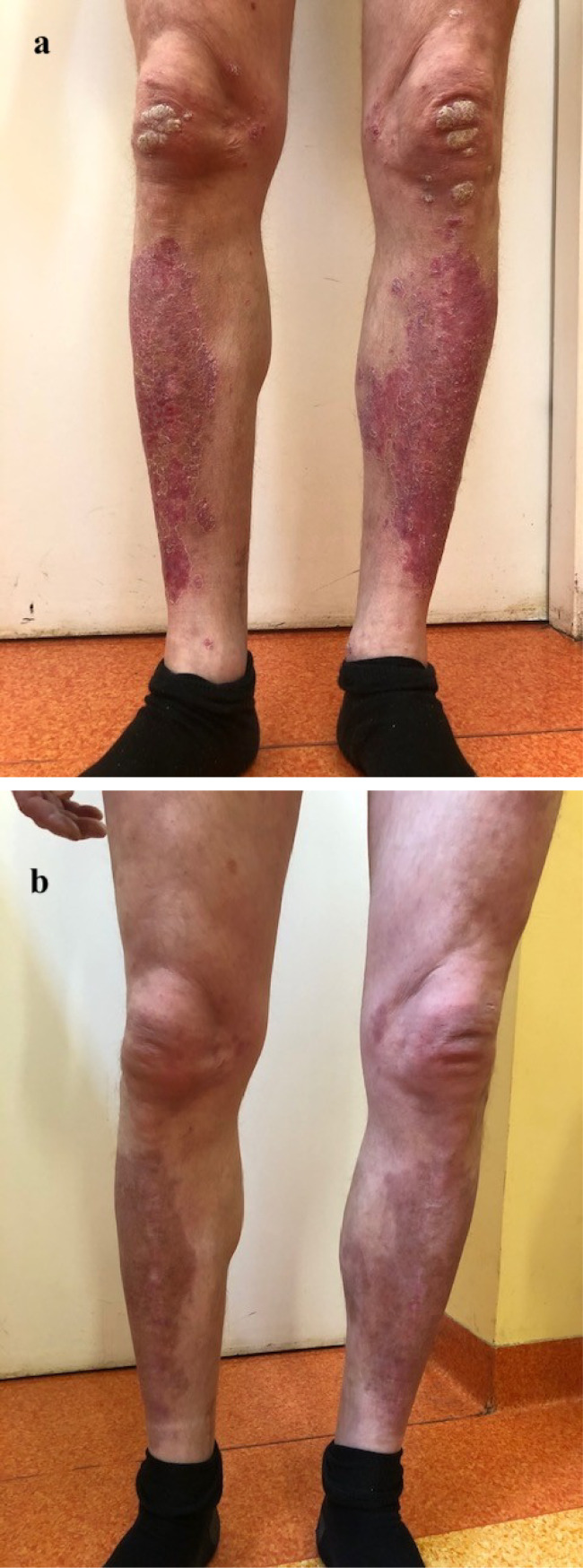
The effects of biologic therapy: **(a)** The patient before the first administration of the biological drug; **(b)** The patient in the course of treatment.

We report a case of a 36-year-old male patient who has been suffering from plaque psoriasis since early childhood, as well as from psoriatic arthritis (PsA) since approximately 30 years of age. He had been most recently treated with secukinumab at a dose of 300 mg weekly for a month since January 2019, followed by a dose of 300 mg once a month. The patient presented to the outpatient clinic in the Department of Dermatology in March 2019 to receive another dose of secukinumab and complained of an asymptomatic pigmented skin lesion on the left calf. On examination, the lesion was approximately 1 cm in diameter and had irregular borders and irregular pigmentation. According to the patient, it had increased in size over the past 3 months. The patient had no personal or family history of melanoma and received no phototherapy in the course of his disease. Previous therapy included infliximab at a dose of 5 mg/kg body weight in weeks 0, 2, and 6 with a maintenance infusion every 8 weeks since May 2018. The treatment was changed to secukinumab due to inadequate therapeutic effects. **Figures 1a, b** show the patient before and during biologic treatment, respectively.

**Figure 2:**
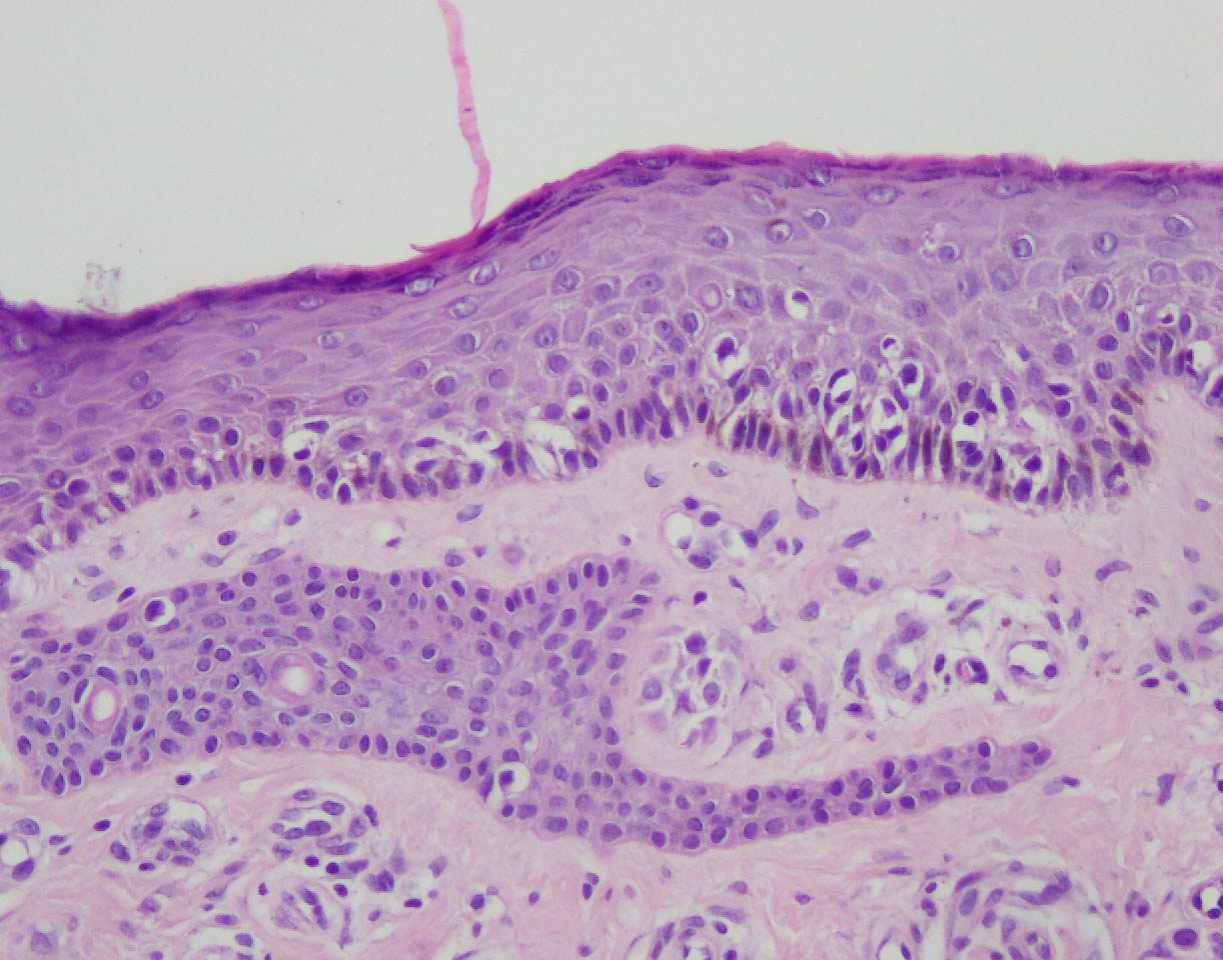
Superficial spreading melanoma with pagetoid scatter of melanocytes, haematoxilin and eosin (H&E) staining, ×200.

The patient was referred for a surgical excision. Histopathological examination of the lesion revealed superficial spreading melanoma classified as pT1a, Breslow 0.6 mm, and Clark II (**Figure 2**). There was no ulceration, vascular, or neural invasion. There were no metastases to local lymph nodes.

We provided the patient with comprehensive information regarding the possible side effects of biologic therapy, including the risk of cancer and the potential for its recurrence after the continuation of the biologic drug. Therefore, the patient was switched to acitretin 35 mg a day and methotrexate 10 mg a week. A year later, the patient observed a new pigmented lesion on the right side of the chest and was therefore qualified for surgical excision. The histopathological examination revealed a compound nevus.

Over two years later, the patient was diagnosed with ulcerative colitis (UC). An endoscopic examination revealed edematous colorectal mucosa, along with ulceration and blurred vascular texture. There were multiple inflammatory polyps along the whole length of the colon. The intestinal mucosal biopsy revealed significant pathological changes, characterized by both acute and chronic inflammatory cell infiltration within the mucosal tissue, accompanied by necrosis and formation of crypt abscesses. These findings suggested ongoing inflammation and tissue damage, indicative of an underlying gastrointestinal condition. Gastric mucosal biopsy revealed no inflammation, metaplasia, or dysplasia. Altogether, the findings indicated an active UC.

Following the diagnosis, the patient was prescribed methylprednisolone at a dose of 4 mg a day by a gastroenterologist.

On the follow-up visit, the patient presented with a single psoriatic lesion on both legs. However, due to the recent appearance of a small amelanotic nodule approximately 0,5 cm in diameter on the patient’s right shin, he was referred for surgical excision, as basal cell carcinoma was suspected. The histopathological examination revealed dermatofibroma.

## 3 Discussion and literature review

Evidence shows that psoriatic patients exhibit a modestly increased risk of overall malignancies, including skin cancers. Chronic low-grade inflammation fosters a tumor-promoting microenvironment, influencing all phases of tumorigenesis and interfering with oncological therapies (13, 17–19). Nevertheless, the relationship between immunomodulatory therapies in psoriasis and the risk of malignancies is complex and still debated. Studies point to elevated oncological risk in patients with psoriasis chronically treated with immunosuppressive agents, including ciclosporin A and methotrexate (7). Importantly, certain researches show that the absolute malignancy risk is insignificant or that the association exhibited inconsistencies when the study cohort was limited to individuals with psoriasis (20, 21).

Numerous cases report malignancies associated with biological drugs, highlighting the need for careful monitoring and evaluation of patients undergoing biological treatment. A 2015 analysis of the FDA Adverse Events Reporting System (FAERS) identified 972 melanoma reports associated with TNF-α inhibitors. Available literature also reports cases of late melanoma recurrence after complete surgical excision linked to the initiation of therapy with etanercept and adalimumab (22).

In our patient, there was a history of biological therapy with infliximab and secukinumab. Infliximab is an antibody binding to and therefore neutralizing TNF-α. Given that TNF-α is a potent pro-inflammatory cytokine, there are significant concerns regarding its potential oncogenic properties (23). A meta-analysis of side effects of the treatment of rheumatoid arthritis with infliximab and adalimumab concluded that both TNF-α inhibitors contribute to an increased risk of severe infections and a dose-dependent higher risk of cancer (24). Contrastingly, another meta-analysis showed similar rates of cancer recurrence among patients receiving conventional immunomodulators, TNF-α inhibitors, a combination of both, or no immunosuppression at all (25). Importantly, vedolizumab and ustekinumab demonstrate similar safety as conventional immunosuppressive treatment in patients with a history of malignancy. Moreover, patients receiving vedolizumab had significantly lower rates of cancer recurrence compared to conventional immunosuppressive drugs (26).

Secukinumab specifically targets IL-17A, a pro-inflammatory cytokine (27). This group of biologics appears to be a safer option for patients with a personal history of cancer, including melanoma. An observational study on 42 psoriasis patients treated with secukinumab for at least 24 weeks and a previous diagnosis of cancer, including 8 cases of melanoma, reported neither tumor recurrence nor progression (28). Analysis of data from 49 clinical trials, including patients receiving secukinumab, demonstrated a low incidence rate of malignancy for up to 5 years of biological therapy, defined as a total of 204 patients per 23908 patient treatment-years (PTY) (29). Another study on a small group of patients with moderate-to-severe psoriasis with a previous history of neoplasms concluded that anti-IL-17 and anti-IL-23 biological drugs can be safely administered in such individuals, describing no cancer recurrence during the mean observation time of 26 months (30).

Notably, the performed analyses assessing the skin cancer risk in individuals undergoing biological therapy do not consider other risk factors of those malignancies, such as phototherapy or lifestyle.

Overall, biological therapies show a comparable safety profile regarding malignancy risk when compared with non-biological drugs. Furthermore, the data provide reassurance for individuals with a history of malignancy, indicating no significant evidence of tumor progression or recurrence associated with such treatments. Despite those conclusions, current clinical practice strongly advises against the continuation of biological therapy in patients diagnosed with malignancies, as an estimated malignancy risk is difficult to evaluate, and its increase due to biologics cannot be completely ruled out. Careful and enhanced skin cancer surveillance is crucial for all patients with autoinflammatory diseases, irrespective of the type of treatment, as evidence suggests an increased general risk of both melanoma and non-melanoma skin cancer (NMSC) in those patients. The immune dysregulation associated with psoriasis and other autoinflammatory conditions is said to be an independent pro-cancerogenic factor (31).

A multidisciplinary approach, including collaboration with oncologists, thorough risk-benefit discussions with patients, and vigilant follow-up, is strongly recommended, especially for those with active or advanced cancer. Future clinical long-term studies are essential to assess the safety of biologic therapies for patients with psoriasis vulgaris and PsA, who have a concomitant malignancy or a history of oncological treatment.

Evidence shows that psoriasis patients are prone to have comorbid inflammatory bowel disease (IBD), including UC. This association is suggested to be a result of concomitant genetic abnormalities, systemic inflammation, and dysregulation in immunological reactions and gut microbiota composition (32). The immunological aspect involves the dysregulated expression of TNF-α and IL-17, among others (33). In our patient, the diagnosis of UC occurred over two years after discontinuation of secukinumab. This timing raises the possibility that the relationship is coincidental rather than a direct result of the biological therapy.

Interestingly, a recent mendelian randomization study suggested IBD to be a causal risk factor for psoriasis and psoriatic arthritis, but not vice versa. However, the pathophysiological mechanisms underlying this phenomenon remain inadequately elucidated. Crohn’s Disease (CD) is particularly linked to the development of both psoriasis and psoriatic arthritis (34). Further research is essential to elucidate the causal relationship between these autoinflammatory diseases.

An induction of IBD in psoriasis can be associated with biological therapy. A paradoxical reaction is either an appearance or worsening of a pre-existing pathological condition that typically demonstrates responsiveness to this pharmacological class, occurring concurrently with the treatment of another disease (35). Among TNF-α inhibitors that can induce IBD, etanercept seems to be the most studied, and numerous cases have been described (36). Importantly, this drug is approved for the management of both psoriasis and PsA, but not for IBD (37, 38).

Infliximab has been utilized in our patient for over three years prior to the diagnosis of ulcerative colitis (UC). As a key agent in the management of UC and Crohn’s disease (CD), infliximab is associated with a negligible risk of inducing inflammatory bowel disease (IBD) in patients with psoriasis (39).

A long-term study on psoriasis patients found no significant correlation between secukinumab and the incidence or exacerbation of IBD (40). Interestingly, one case study reports a paradoxical UC in a psoriasis patient after a 4-month therapy with secukinumab. Complete remission of UC was achieved after secukinumab withdrawal and the introduction of adalimumab (41). In our patient, there was an almost 3-year interval between the cessation of secukinumab treatment and the subsequent UC diagnosis.

## 4 Conclusions

Our case report highlights the challenges associated with managing malignancies in patients undergoing biologic therapy, underscoring the importance of thorough medical assessment and examination for individuals receiving these treatments. In instances of coexisting cancer, a multidisciplinary approach is essential for effectively addressing both oncological and autoinflammatory diseases. Furthermore, healthcare professionals should be alert to the increased risk of developing other autoinflammatory conditions in patients with psoriasis. To better characterize the cancer risk profile in patients using different classes of biologics, long-term prospective studies are essential.

## Data Availability

The original contributions presented in the study are included in the article/supplementary material. Further inquiries can be directed to the corresponding author.
